# Characterization of non-invasive oropharyngeal samples and nucleic acid isolation for molecular diagnostics

**DOI:** 10.1038/s41598-024-54179-6

**Published:** 2024-02-19

**Authors:** Leonie Hose, Matthias Schürmann, Inga Mennebröcker, Rayoung Kim, Tobias Busche, Peter Goon, Holger Sudhoff

**Affiliations:** 1 Department of Otolaryngology, Head and Neck Surgery, Campus Klinikum Bielefeld Mitte, University Hospital OWL of Bielefeld University, Teutoburger Str. 50, 33604 Bielefeld, Germany; 2 Center for Biotechnology (CeBiTec), University Hospital OWL of Bielefeld University, Bielefeld, Germany; 3 Department of Medicine, Yong Loo Lin School of Medicine, National University of Singapore, National University Health System, Singapore, Singapore

**Keywords:** Molecular medicine, Transcriptomics

## Abstract

Molecular diagnostics is an increasingly important clinical tool, especially in routine sampling. We evaluated two non-invasive methods (oral swabs and mouthwashes) for sampling nucleic acids from the oral/pharyngeal area. We created a workflow from sample collection (n = 59) to RT-qPCR based analysis. The samples were further characterized in terms of their cellular composition as well as the purity, degradation and microbial content of the derived DNA/RNA. We determined the optimal housekeeping genes applicable for these types of samples. The cellular composition indicated that mouthwashes contained more immune cells and bacteria. Even though the protocol was not specifically optimized to extract bacterial RNA it was possible to derive microbial RNA, from both sampling methods. Optimizing the protocol allowed us to generate stable quantities of DNA/RNA. DNA/RNA purity parameters were not significantly different between the two sampling methods. Even though integrity analysis demonstrated a high level of degradation of RNA, corresponding parameters confirmed their sequencing potential. RT-qPCR analysis determined *TATA-Box Binding Protein* as the most favorable housekeeping gene. In summary, we have developed a robust method suitable for multiple downstream diagnostic techniques. This protocol can be used as a foundation for further research endeavors focusing on developing molecular diagnostics for the oropharyngeal cavity.

## Introduction

Molecular diagnostics, a cornerstone of modern medicine, usually necessitates samples drawn via blood venepuncture or tissue biopsy. Medical professionals are needed to take the samples and invasive methods are often uncomfortable for patients. Therefore, the development of non-invasive techniques for sample acquisition is important to speed up clinical diagnosis and a promising tool for biomarker identification in the scientific community without direct access to medical professionals. This non-invasive sampling might give insight into diseases localized to the region of sampling, e.g. in the mouth but also for general systemic health circumstances. Particularly for diseases of the mouth, numerous studies on biomarker identification for molecular pathogenesis in saliva have already been published. These include potential candidates for gingivitis and periodontitis, which are major oral health threats^1,2^, but also Sjögren´s syndrome, an autoimmune disease that attacks the salivary and lacrimal glands as well as oral cancer^3^. Additionally, biomarkers for bacterial, viral and fungal infections have been reported^4^. In addition to saliva, swabs and mouthwashes may be important to obtain mucosal-, bacterial- or immune cells. The difference in sampling between saliva and mouthwash is, that saliva is collected directly into a vessel. In the case of mouth washing, the oral cavity is rinsed with NaCl and cells are mechanically removed. In this context it has already been possible to take oral mucosa cells using various swabs and to isolate nucleic acids from this material^5^. For this, the brush swab was proven to be advantageous compared to for example wooden spatula or cotton swab spatula^5^. It has also already been possible to obtain cells for DNA isolation from mouthwashes and it has been proven to be a particularly simple method to collect biological material for diagnostics, as this does not even require input from medical professionals ^6–8^. In order to be able to carry out molecular diagnostics, the yield as well as the quality of the isolated DNA and RNA play an important role. This is why a well-established and reliable protocol of nucleic acid extraction is essential for further analysis. Molecular diagnostic tests are used with the aim of identifying diseases, determining the cause, identifying individual predisposition and assessing response to therapy^9^. Examples of well-established molecular diagnostic methods are quantitative "real-time" PCR, melting point analysis for the detection of polymorphisms, multiplex PCR and long range PCR^10^. However, for diseases closely coupled to gene expression, e.g. for inflammatory processes or pathogen detection (viruses), RNA analysis is an obvious choice. In addition, RNA analyses offer the possibility of analysis at the transcriptional level, and can therefore be used for biomarkers in cancer diagnosis. RT-PCR-based diagnostics has many advantages, which is why it has found a permanent place in laboratory diagnostics. For example, it is quick and easy to perform and has low cost implications, an increased ability to detect low-level microorganisms (viruses, bacteria)^11^. For standardization, different housekeeping genes (HKGs) can be used, for example *GAPDH, YWHAZ, ACTIN-B, 18srRNA, TBP, B2M* and *HMBS*, which were already used in human samples^12,13^. Software with various algorithms like geNorm, BestKeeper, NormFinder and RefFinder are freely available for assessing stability of different HKG^14,15^. In many laboratories, Frederick Sanger`s classical DNA sequencing technique has been utilized for molecular diagnostics in addition to PCR analyses. Increasingly, however, the relatively new next generation sequencing (NGS) is gaining traction despite its associated challenges. It is and has been used in research, but the transformation to a diagnostic tool has already taken place in some diagnostic laboratories and will continue in the near future due to many advantages^16^. Therefore, quality control and sample characterizations are essential for this purpose. There are some quality parameters, like Q30 and per base sequencing, that should be considered and simple tools (FastQC) that allow you to assess the quality of the measurement performed before evaluations are made ^17^. RNA-Seq analysis offers both the quantification of known or predefined RNA species and the ability to detect and quantify rare and new RNA transcript variants within a sample, enabling the detection of new biomarkers^18,19^. An interesting example is mRNA expression profiling using RNA-Seq for cancers. In breast cancer, recent clinical guidelines support the use of mRNA-based prognostic tests for multiple genes^20^. Potential RNA markers that would be interesting for RNA-Seq analyses in oral tumors have also been found in saliva^21^. These would be interesting candidates for the diagnosis of oral and oropharyngeal tumors. All in all, RNA-Seq has the potential to revolutionize clinical testing for a wide range of diseases. Once the discovery phase is complete, many diagnostic tests will become targeted tests that are sensitive enough to detect a small number of rare transcripts^22^. Due to the previously mentioned possibilities of molecular analysis of nucleic acids from non-invasive oral samples, we describe and compare two methods of sampling from the human oral cavity and nucleic acid extraction quantitatively and qualitatively in this study. The aim was to develop a consistent and reliable method using non-invasive material sampling for molecular downstream analysis and to describe the advantages as well as limitations and difficulties. We focus on human mRNA, highlighting its advantages, limitations, and challenges. Our emphasis is on human mRNA, given its potential for diagnosing oral conditions such as carcinoma, inflammation, and various other diseases.

## Material and methods

### Participant consent and study design

**Figure 1:**
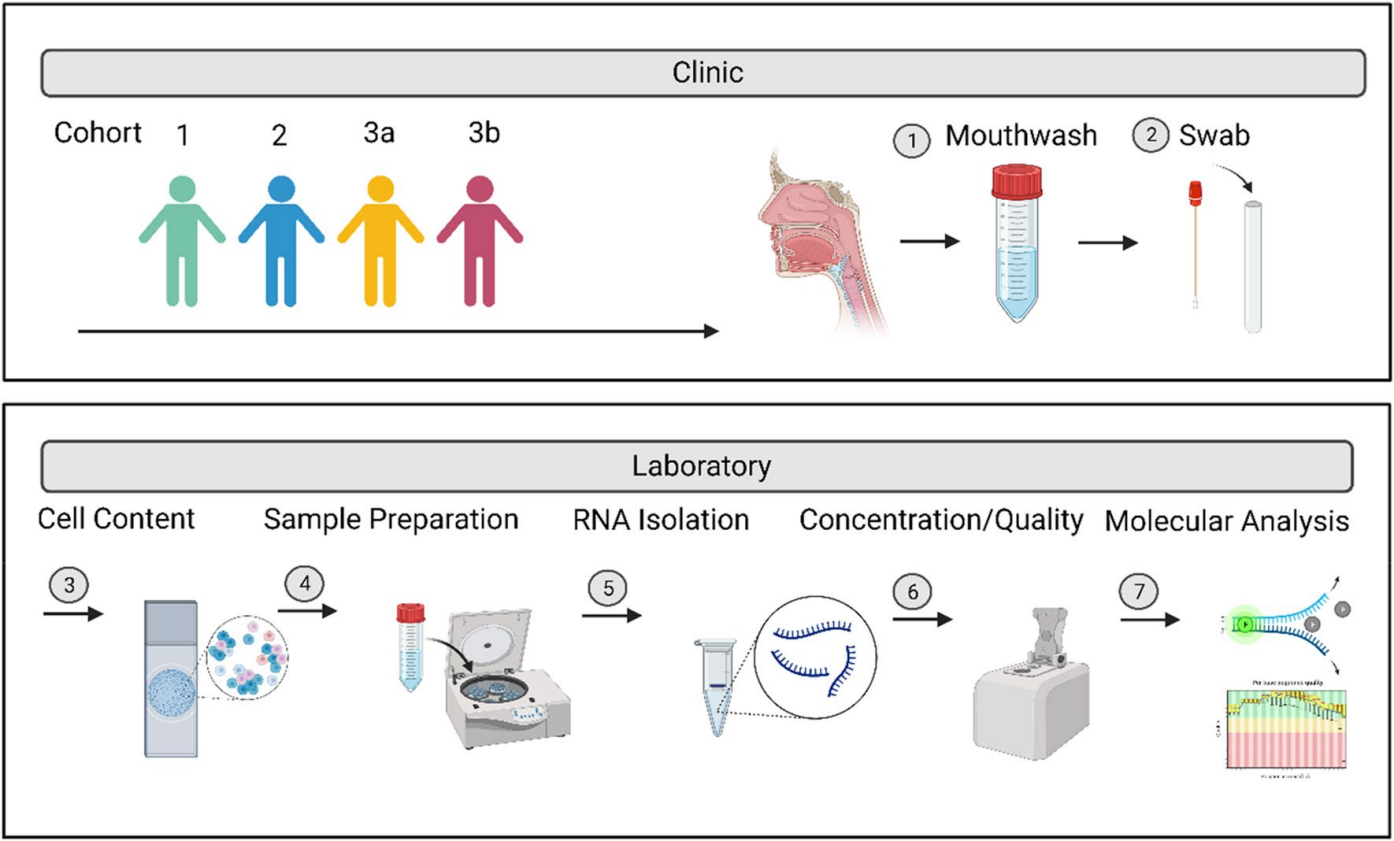
Study design and procedure in clinic and laboratory.

This study was approved by the Ethics Committee of the university hospital Ruhr-Universität Bochum in Bad Oeynhausen, Germany (2022_060_1). All participants gave written informed consent according to the agreed patient information sheets. All methods were carried out in accordance with relevant guidelines and regulations. Sample collection took place in the outpatient clinic of the ENT department of the university hospital Bielefeld Mitte (Fig. 1).

### Study design and procedure in clinic and laboratory

This study was divided into the clinic and laboratory sections. The clinic part consists of amassing the cohort by selecting and educating suitable patients. In addition, the sampling of mouthwashes and swabs takes place in the clinic. In the laboratory samples are processed and nucleic acids extracted and tested for quality and quantity. Subsequently, molecular biological analyses are carried out.

The study cohort is composed of different groups of patients, depending on their current stage of disease. These are described in the Table 1.

**Table 1:** Study cohort.

n = 59 41 males (m) 18 females (f)	Group 1 (n = 13) 11 m 2 f	Patients with symptoms that are not due to carcinoma
	Group 2 (n = 8) 6 m 2 f	Patients with high tobacco/alcohol consumption and/or symptoms of carcinoma
	Group 3 (n = 38) 24 m 14 f	a: Acute tumor—oropharyngeal carcinoma (n = 7)
		b: Post diagnosis treatment (n = 31)

Overview of the study cohort and patient grouping. Shown are the numbers of included patients, sex and grouping depending on the symptoms and status.

### Sample collection

For mouthwash collection a 20 ml 0.9% NACL solution is handed out. This is used to rinse and gargle thoroughly in the mouth and throat area for 30 s. A professional tonsil swab is then performed by a physician with a Gynobrush^®^ (Heinz Herenz Medizinalbedarf). For tumor patients, the tumor region is swabbed directly. The swab is then placed in an Eppendorf tube filled with 300 µl 1× RNA Shield (Zymo Research, Europe GmbH) for protection. The samples are cooled on ice until sample preparation proceeds. Henceforth, swab = Gynobrush sampling.

### Sample preparation and nucleic acid extraction

**Figure 2:**
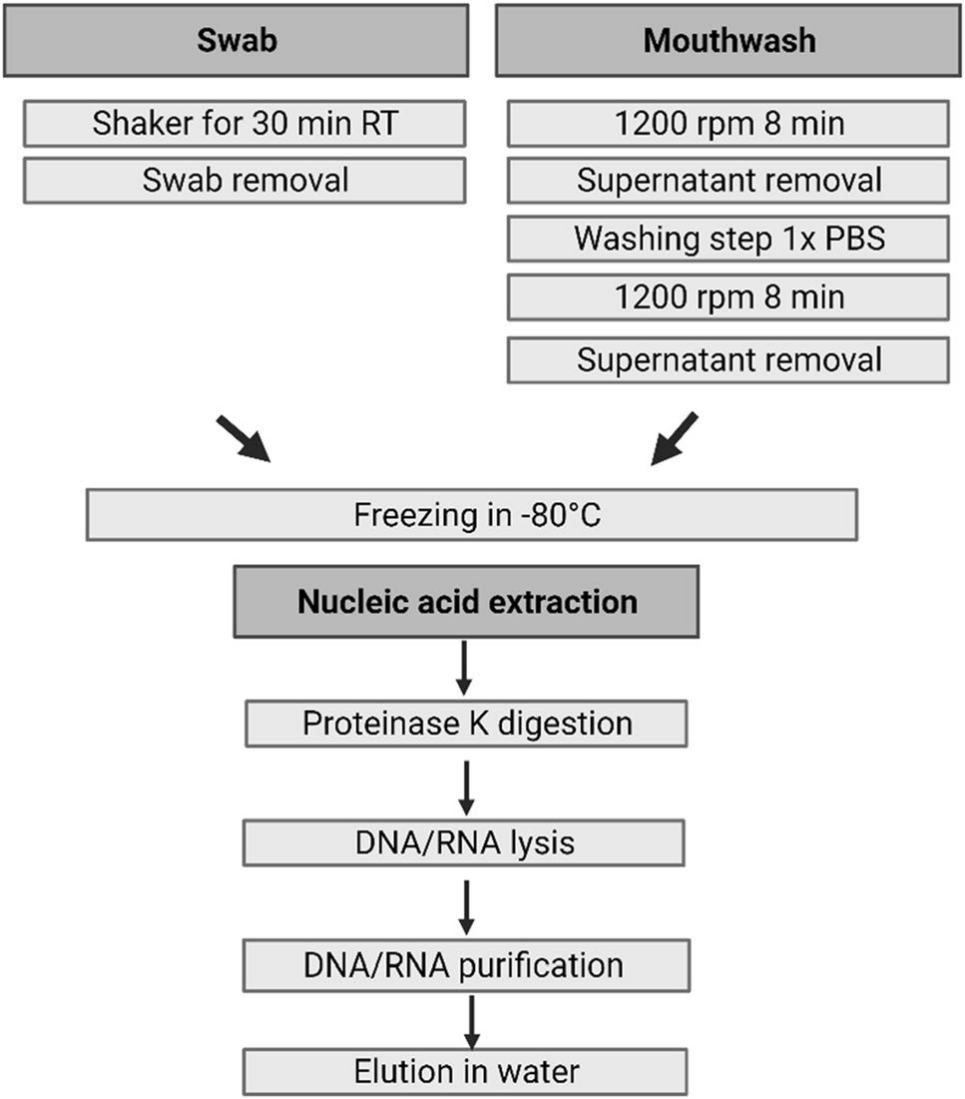
Sample preparation and nucleic acid extraction.

We have made several refinements to the procedures that follow sample collection in the clinic. This text outlines the original isolation and preparation method and the Table 2 shows the optimized steps in comparison to the original protocol**.** Figure 2 shows the final sample preparation after optimization.

**Table 2:** Optimized protocol.

DNA/RNA isolation	Before optimization	After optimization
Sample prep	No washing step	Washing with 1× PBS
Digestion	Proteinase K digestion for 30 min. at RT	Proteinase K digestion for 1 h at 50 °C
Purification	–	–
Elution	Elution in 35 µl nuclease free water	Elution in 20 µl nuclease free water

Overview of the optimized protocol steps in direct comparison to the original protocol.

After sample collection in the clinic, the samples are stored on ice from collection until processing in the laboratory. In the laboratory, the mouthwash is centrifuged at 1200 rpm for 8 min. The supernatant is pipetted off. The cell pellet was frozen down at −80 °C for storage. The swabs, stored in RNA Shield, are placed on a shaker for at least 30 min to release the cells from the swab brush. After that, the swab brush is removed and the samples are stored at −80 °C until nucleic acid isolation.

For DNA and RNA extraction the samples are thawed and the cell pellet from the mouthwashes are dissolved in 300 µl 1× RNA Shield. DNA and RNA from all mouthwash and swab samples was extracted using the Quick DNA/RNA Microprep Plus Kit (Zymo Research, Europe GmbH). The protocol was carried out in three steps and DNA and RNA extraction is performed in parallel.

#### (1) Digestion

Proteinase K digestion is performed with a total volume of 300 µl of sample. Before optimization, the incubation time for digestion was 30 min at RT. Lysis Buffer is then added to the sample in a ratio of 1:1.

#### (2) DNA and RNA purification

The purification first runs over a column that binds DNA. RNA is left and is mixed in a ratio of 1:1 with 95% ethanol. The sample is then pipetted onto a second column, which is used for RNA binding. To completely remove DNA from the samples, a DNase treatment is carried out with all RNA samples. For this, a mix of DNase and DNA digestion buffer is added to the column and incubated for 15 min at room temperature. Then both columns are washed with prep and wash buffer.

#### (3) Elution

Samples were eluted in 35 µl nuclease free water.

### Concentration and quality analysis

RNA was quantified after extraction by measuring absorbance using the Nanophotometer^®^ (IMPLEN) and A260/A280 and A260/230 ratios were measured for quality assessment. The RNA was then analyzed with an RNA Screen Tape kit on the Tape-Station. The RIN values were calculated with Tape Station Analysis Software 4.1.1 (Agilent Technologies). To address the challenges of the oral RNA, the DV200 was calculated as well. All RNA samples were stored at −80 °C and all DNA samples at −20 °C for further analysis.

### cDNA synthesis

For comparability of further analyses, all RNA samples are adjusted to a concentration of 20 ng/µl. cDNA synthesis is then performed with the cDNA synthesis kit—all priming options (Biozym Scientific GmbH). The synthesis is carried out with random hexamer primers and was performed according to the manufacturer’s instructions.

### RT-qPCR

Housekeeping gene analysis was performed by RT-qPCR. For all RT-qPCR reactions a ready to use master mix (Luna Universal qPCR Mix, New England Biolabs) was used. All measurements were run in triplicate with a total volume of 10 µl each and the cycling conditions were as follows:

Step 1—35 °C for 25 min.

Step 2—95 °C for 3 min.

Step 3—(40 repeats).

    95 °C for 15 s.

    60 °C for 30 s.

Step 4—50 °C to 95 °C at 0.1 °C/s (Meltingcurve acquisition).

### Primer design

For the RT-qPCR analysis seven different housekeeping gene (HKG) candidates were chosen. *18srRNA, YWHAZ, TBP, B2M, TFRC, ACTB* and *HMBS* sequences were selected with Ensemble Genomebrowser (Ensembl genome browser 111). In addition, a primer covering the variable region 6 (V6) of the *16srRNA* gene was designed for detection of bacterial RNA. Primers were designed and verified with the NCBI Primer Tool (Primer designing tool (nih.gov)). All HKG primers and *16srRNA* primer as well as the target gen *BACE2* are listed in Table 3, with corresponding sequences. For HKG analysis, the freely available software NormFinder (NormFinder (moma.dk), 2004) ^14^ was used.

**Table 3:** Primer sequences.

Gene name	Gene symbol	Forward (5′-3′)	Reverse (3′-5′)
*18s ribosomal RNA*	*18srRNA*	GGTGGTGCCCTTCCGTCA	CGATGCGGCGGCGTTATT
*Tyrosin-3-monooxygenase-activation protein, zeta polypeptide*	*YWHAZ*	GCCCACCCATTGTCCCC	TTATGGCTCGGAAACGGGAG
*TATA-binding protein*	*TBP*	TGGCGTGTGAAGATAACCCAA	CGCTGGAACTCGTCTCACTA
*Beta-2-Microglobulin*	*B2M*	TTGAGTGCTGTCTCCATGTTTG	TCTGCTCCCACCTCTAAGT
*Transferrin receptor*	*TFRC*	GACACGTCTGCCTACCCATT	CCGTTTCCAACTGCCCTATG
*Beta-actin*	*ACTB*	CCCTGGACTTCGAGCAAGAG	AAGGAAGGCTGGAAGAGTGC
*Glyceraldehyd-3-phosphate dehydrogenase*	*GAPDH*	CTGCACCACCAACTGCTTAG	GTCTTCTGGGTGGCAGTGAT
*16s ribosomal RNA*	*16srRNA*	TCGATGCAACGCGAAGAA	ACATTTCACAACACGAGCTGACGA
*Beta-secretase 2*	*BACE2*	TAACGCAGACAAGCCATCG	CCACCGCATCAAACACCTTC

Overview of analyzed housekeeping genes and their forward and reverse primer sequences.

### RNA sequencing analysis

Poly A-selected libraries were prepared from 200 ng of total RNA using QuantSeq 3′mRNA-Seq Library Prep Kit FWD for Illumina (Lexogen), according to manufacturer's instructions. Size distribution and quality of the libraries were assessed by Tape Station Analysis Software 4.1.1 (Agilent Technologies) and final libraries were sequenced 75 bp single-end mode on a NextSeq2000 with a 3 chemistry.

### Cytology, staining and cell counting

Three cytological preparations for both methods were made for microscopy. The swab brushes were rolled directly onto slides and the mouthwashes were prepared using a cytocentrifuge. A 40 µm cell strainer was used to prepare leukocyte-free preparations. Cytoslides were stained using a Papanicolaou´s stain at the Department of Pathology, Klinikum Bielefeld. Light microscopy was performed at a 200× magnification. The observers moved through each slide until epithelial cells (min 50 cells in total) from at least three fields of view had been counted. The count was performed in double determination by two observers. Epithelial cells were counted and differentiated as intermediated cells, or superficial. The cell numbers of both observers were then averaged and given as a percentage.

### Statistical analysis

The data was summarized in Microsoft Excel 2021 and then GraphPad Prism 8 software was used for graphical plotting and analyses. The differences between swabs and mouthwashes were tested by functions of unpaired, two-tailed t-test. The quantified data were presented as mean and ± standard deviation.

## Results

**Figure 3:**
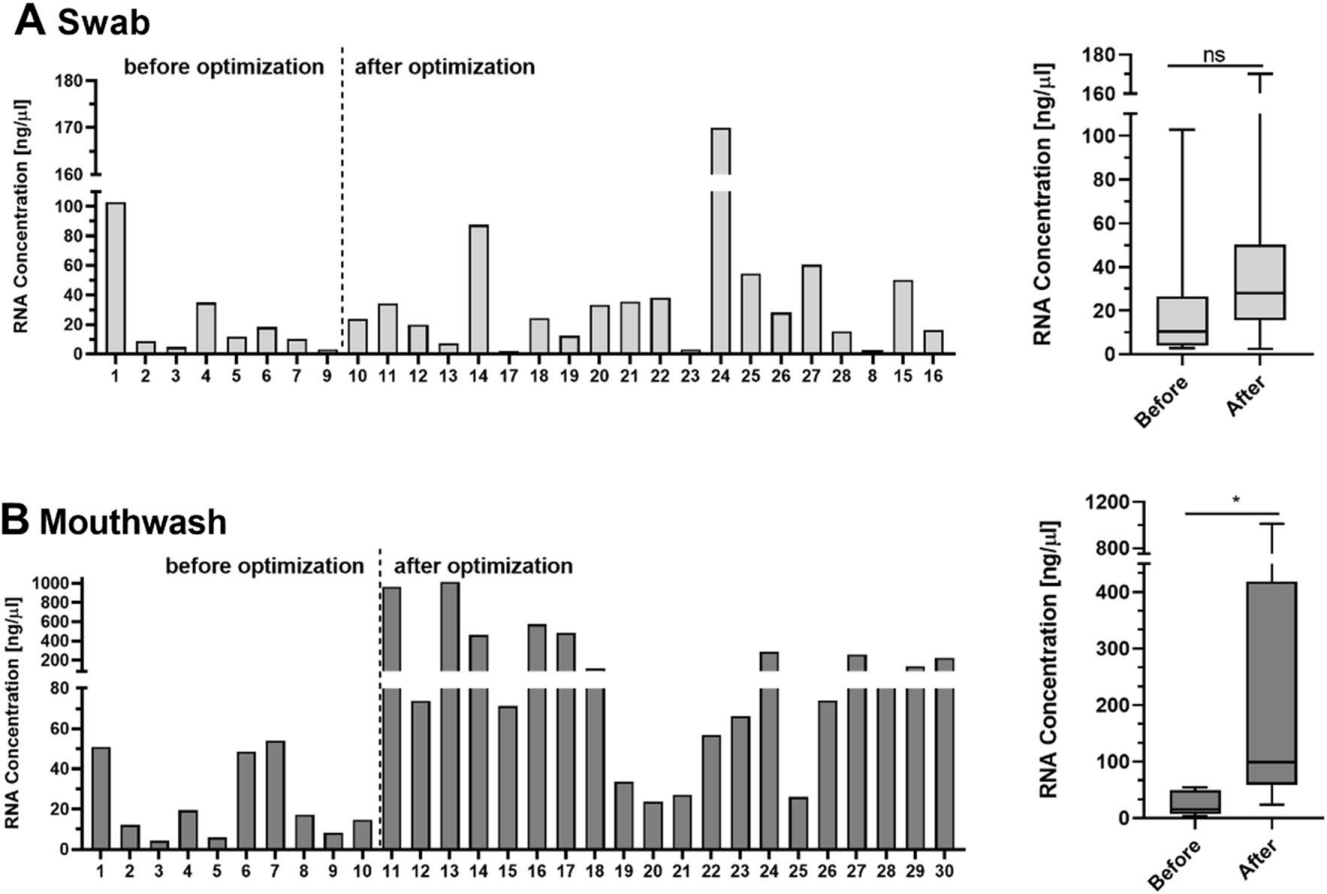
Comparison of protocol optimization for RNA samples. The bars on the left side in (**A**) show the RNA concentration of swab samples in ng/µl. The Figure shows the concentrations before and after protocol optimization. The boxplots on the right side of (**A**) show same samples concluded into a boxplot for before and after. There is no significant difference between the boxplots (t-test, two-tailed, 95% confidence interval, *p < 0.05, **p < 0.01). Same graphs are shown for mouthwash samples below in (**B**). There is a significant difference (*p = 0.0242) between before and after optimization. The concentrations after optimization are 10 times higher than before.

As previously described**,** the protocol was optimized using ten samples. As mentioned, the conditions of the proteinase K digestion were optimized and the elution volume was also reduced as well as a washing step of the cell pellet in PBS was added for the mouthwashes. The changes to the protocol resulted in a better RNA yield, especially for the mouthwashes, shown in Fig. 3. The concentrations before and after optimization show a significant difference on average (*p = 0.0242) for mouthwashes (Fig. 3B). An increase in RNA concentration by a factor of 10 could be achieved. Although a slight improvement by a factor of about 1.7 in RNA yield was also achieved with the swab samples (Fig. 3A), but this did not reach statistical significance (*p* = 0.2963). In due course, only samples after optimization were used for concentration and quality analysis.

### Comparison of protocol optimization

#### Nucleic acid concentration

**Figure 4:**
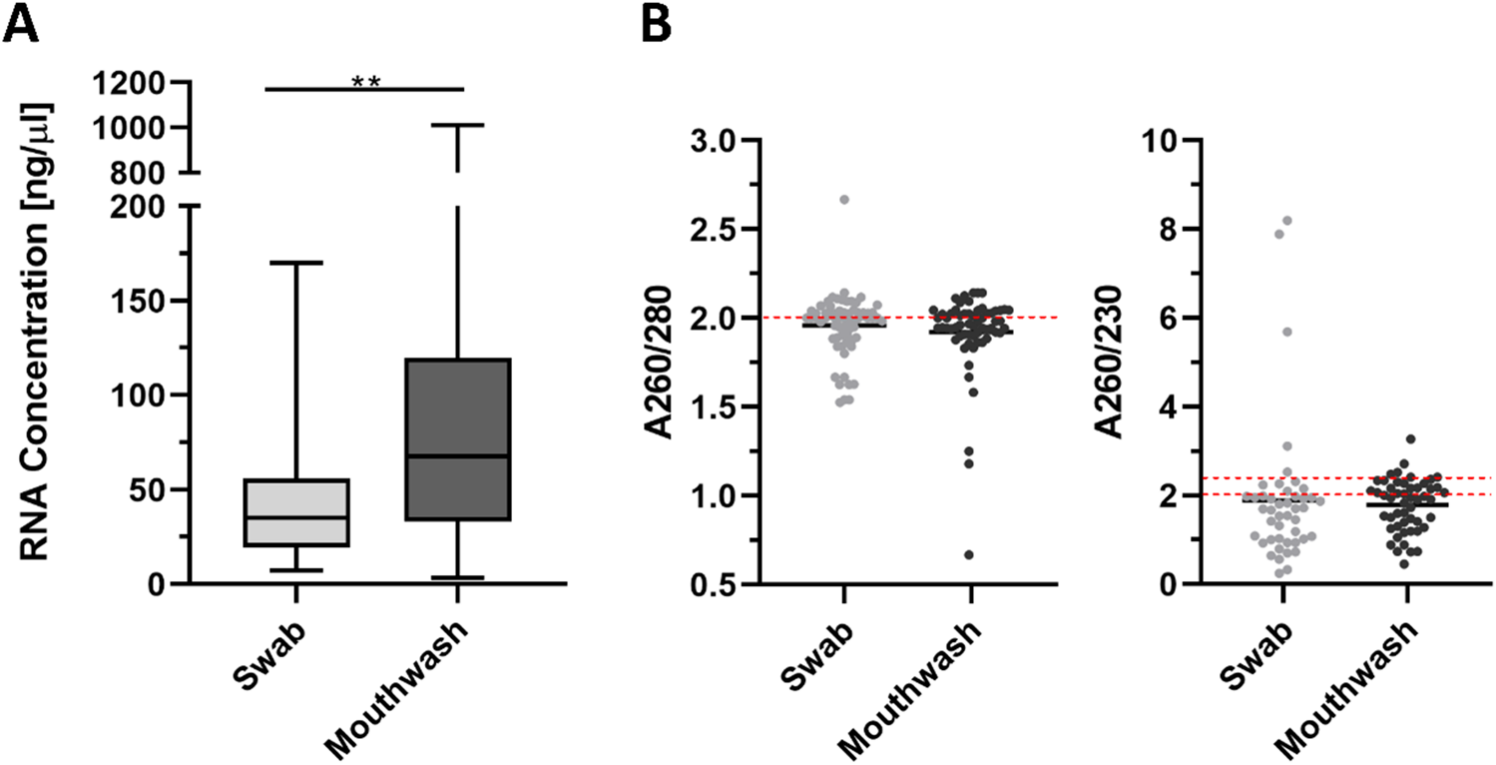
Molecular properties of RNA. The boxplots in (**A**) show the amounts of total human and bacterial RNA in µg/µl extracted from the swab (n = 46) and mouthwash (n = 50) samples. There is a significant difference between swab and mouthwash RNA concentration (**p = 0.0025), (t-test, two-tailed, 95% confidence interval, *p < 0.05, **p < 0.01). Quality of extracted RNA is shown in (**B**) for comparison, using the A260/230 and A260/280 ratios. Red lines show the area of “pure” nucleic acid. Swab and mouthwash samples show concerning values. While the desired range of 2.0–2.2 for alcohol and salt contamination is slightly undershot by both methods, the desired value of 2.0 for protein contamination is almost achieved on average by both methods.

Yields averaging 144.1 ng/µl (SD ± 214.9) total RNA were achieved with the 2 ml mouthwashes. In comparison, concentrations of 44.52 ng/µl (SD ± 34.14 ng/ul) total RNA on average were achieved with the swabs. As shown in Fig. 4A there is a statistically significant difference of the RNA concentration between the two sampling methods. Mouthwashes show a significantly higher RNA concentration (*p = 0.0025) than swab samples. For DNA, a mean value of 46.82 ng/µl (SD ± 26.98) was isolated for the mouthwashes and in comparison, 32.77 ng/µl (SD ± 20.99) DNA was isolated in the swabs (Suppl. Fig. 3A). A significant difference between the sample methods could also be demonstrated in Suppl. Fig. 4.

### RNA quality

The quality measurement of the RNA and DNA is divided into the absorption measurements at A260/280 and at A260/230. The comparison of swab and mouthwash shows similar results for protein contamination (A260/280) and contamination with organic substances (A260/230). The measured values are shown in Fig. 4B and the target values and ranges are marked with red lines. The swabs reach on average 1.959 for A260/280 and 1.888 for A260/230. The mouthwashes also depict a ratio of 1.921 for A260/280 and 1.784 for A260/230. Thus, the mean values of both methods for protein contamination are approximately at a desired value of 2.0. The mean value for organic substance impurities is below the undesirable value of 2.0–2.2 for both methods. The comparison of swab and mouthwash also shows similar values for DNA. It is noticeable that the contamination by organic substances or salt in the DNA of both sample materials is stronger than in the RNA. On average, for the DNA, the swabs achieve 1.775 for A260/280 and 1.592 for A260/230. The mouthwashes also show a ratio of 1.767 for A260/280 and 1.428 for A260/230 (Suppl. Fig. 4B). We also did a RIN analysis, which represents RIN values of 3.3–5.3 for the swabs and 1.1–3.6 for the mouthwash (Supp Fig. 4). Due to the low RIN values, a DV200 determination was carried out for further quality measurements and analysis of the size distribution of the fragments. Values between 57 and 98% could be generated with both methods (except for an outlier of 7% for the swabs, raw data not shown).

### Comparison of housekeeping gene expressions

Firstly, we selected *GAPDH* and found that it showed a non-specific double peak in the melting curve with the mouthwash samples. Testing the same primer for *GAPDH* with the swab sample of the same healthy patient and in addition a pure human cell culture showed a single melting peak (Suppl. Fig. 8). To find the best possible HKG for our methods we started an HKG analysis with seven different genes (*ACTB, TFRC, YWHAZ, HMBS, B2M, TBP, 18srRNA*) for each method (swab n = 7, mouthwash n = 9). For this purpose, a homogeneous distribution of samples before and after optimization is tested in order to test the RT-qPCR method on all samples. The means and standard deviation of raw Ct-values are shown in Table 4. The lowest Ct-values are achieved with the gene *18srRNA* (swabs: mean 12.4, SD ± 1.763, mouthwash: mean 13.07, SD ± 2.52), while the highest Ct-values are achieved with the gene *HMBS* (swabs: mean 24.78 SD ± 3.641 mouthwash: mean 26.38, SD ± 2.117). Basically, very similar Ct-values were measured with both methods and no important difference can be found in the number of cyclic genes reached.

**Table 4:** Overview of raw Ct-value statistic of the HKG analysis.

	*ACTB*		*TFRC*		*YWHAZ*		*HMBS*		*B2M*		*TBP*		*18srRNA*	
	Mean	SD ±	Mean	SD ±	Mean	SD ±	Mean	SD ±	Mean	SD ±	Mean	SD ±	Mean	SD ±
Swab	18.08	1.998	25.24	2.433	25.62	1.485	24.78	3.641	22.30	1.857	25.13	1.864	12.40	1.763
Mouthwash	17.64	3.067	24.01	1.712	24.96	1.437	26.38	2.117	22.11	5.119	25.00	1.822	13.07	2.52

This table summarizes the raw Ct-values of all tested HKGs including their standard deviation for the swabs and the mouthwashes.

**Figure 5:**
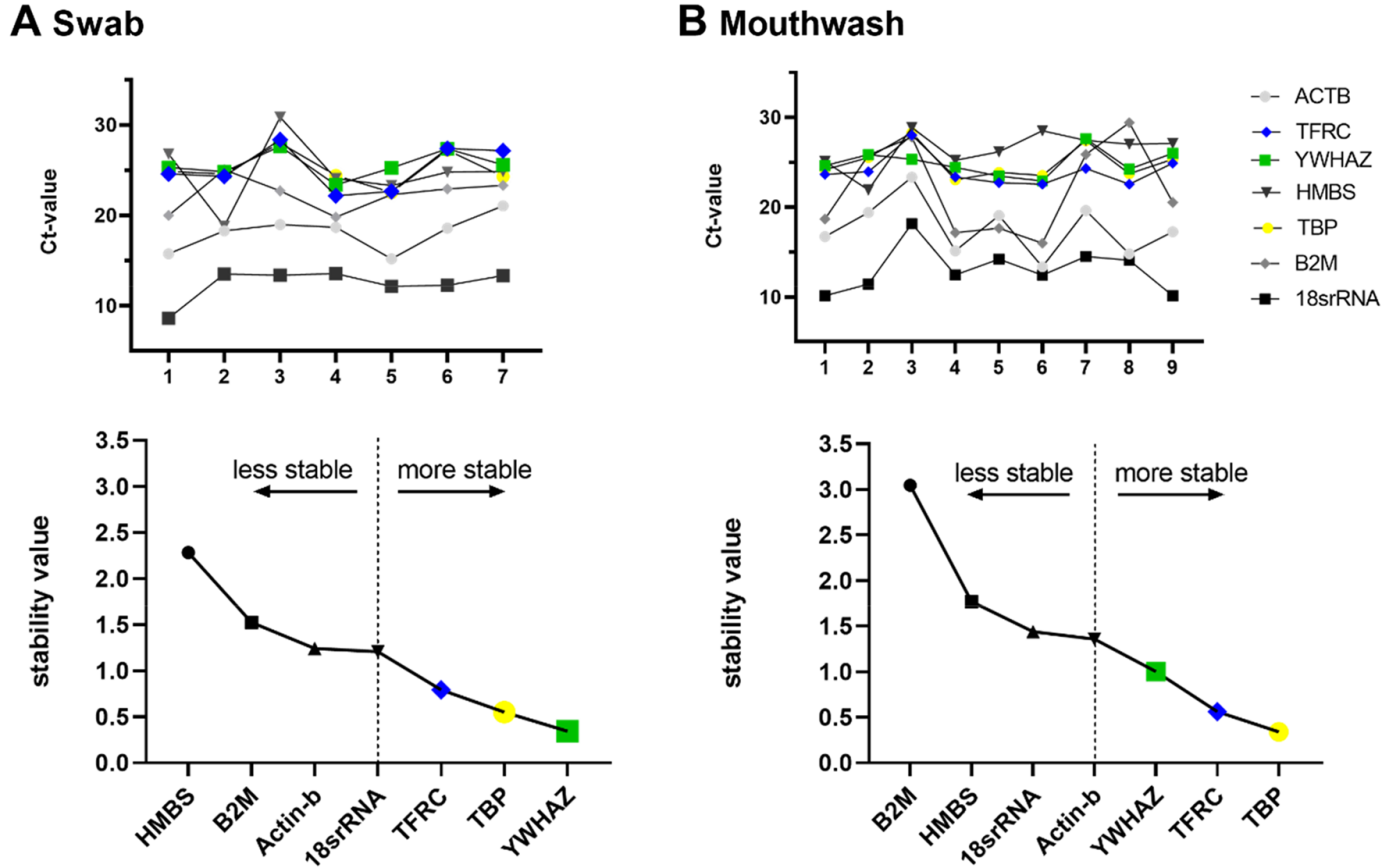
Housekeeping gene analysis with NormFinder. This figure shows a graph with the raw Ct-values of HKGs (*ACTB, TFRC, YWHAZ, HMBS, B2M, TBP, 18srRNA*) and underneath HKG ranking based on stability values. These values were calculated with the algorithm NormFinder and indicate the quality/stability of the housekeeping genes for the respective sample method. (**A**) shows the HKG analysis for the swabs and (**B**) for the mouthwashes. In (**A**) and in (**B**) are the same best three genes shown for both methods, but their order differs. For swab samples the gene *YWHAZ* with a stability value of 0.342 is the best gene and for the mouthwash samples the gene *TBP* shows the lowest value with 0.339.

To assess the stability of the genes and to find the best genes for each method, an analysis was performed using NormFinder. Figure 5A shows the raw Ct-values and underneath the stability values of the swab samples. For comparison, similar graphs are shown for the mouthwashes in Fig. 5B. The measure of stability is given as the stability value, which are inversely proportional to the actual stability of the gene.

### Housekeeping gene analysis

The best three genes for both methods are *TFRC, TBP* and *YWHAZ*. However, their order differs between the methods, so that *YWHAZ* with a value of 0.342 is the most stable for the swabs, followed by *TBP* (0.551) and *TFRC* (0.793). For the mouthwashes, *TBP* with a value of 0.339 is the gene with the lowest stability value, followed by *TFRC* (0.561) and *YWHAZ* (1.003). The highest and thus worst stability value among the swabs was achieved by the *HMBS* gene, with a value of 2.282. For the mouthwashes, the worst stability value of 3.046 was measured with the *B2M* gene. Loading all modified Ct-values to NormFinder considering a grouping of swab and mouthwash resulted in *TBP* as the best common HKG with a stability value of 0.161.

**Figure 6:**
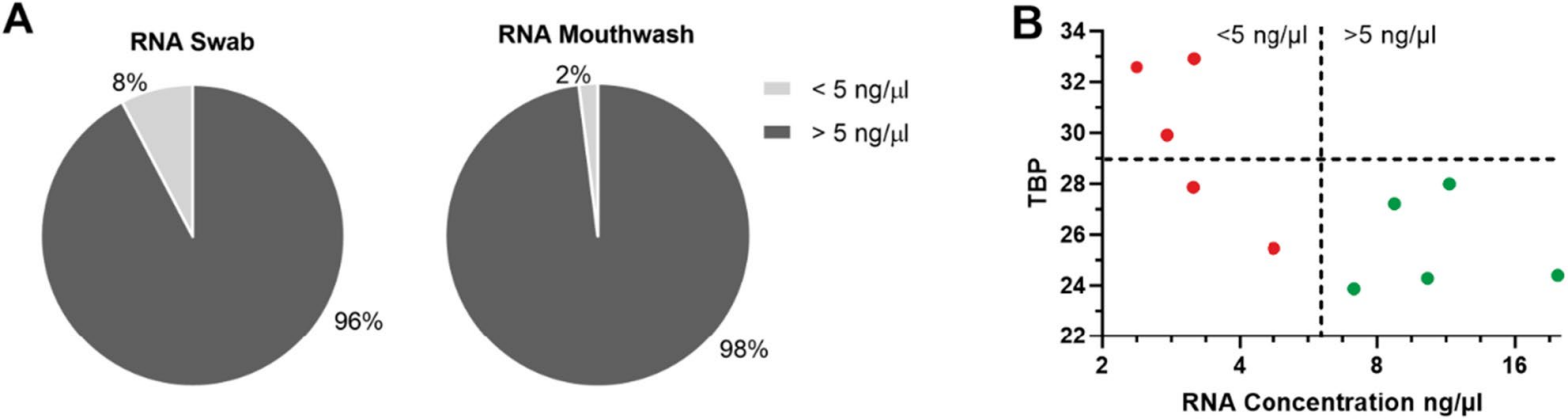
Low RNA concentration and qPCR Limits. The circle-chart in (**A**) shows the percentage of RNA concentration < 5 ng/µl (light grey) and > 5 ng/µl (dark grey) in swab and mouthwash samples. While 8% of swabs are under 5 ng/µl, only 2% of mouthwashes are under 5 ng/µl. The plot in (**B**) shows the raw Ct-values vs. RNA concentration of qPCR measurements with the gene *TBP*. n = 5 samples (in red) represent swab samples with a concentration < 5 ng/µl and are compared with n = 5 swab samples (in green) with a concentration > 5 ng/µl. The samples under < 5 ng/µl show significantly higher Ct-values (red) than the samples < 5 ng/µl (green) (p = 0.0004). The mean value of all red values is 31.07 and the mean value of the green samples is 25.62. Especially three red samples show significantly higher Ct-values. These are already in the negative control (water) range. Two of the red samples shows a similar value to the green samples (27.69).

As can be seen in Fig. 6A, 6% of the RNA samples of the swabs and 2% of the mouthwash samples show concentrations < 5 ng/µl (n = 50). For DNA samples, there are 24% < 5 ng/µl for the swabs and 8% < 5 ng/µl for the mouthwashes (n = 50) (Suppl. Fig. 10). To check the applicability of these low concentration RNAs for RT-qPCR analysis, we analyzed them with *TBP*, the most suitable gene for the swabs and mouthwash, using RT-qPCR (Fig. 6B). In red, the raw Ct-values of the samples with < 5 ng/µl are shown (n = 5) in comparison to the green samples with > 5 ng/µl (n = 5). Three of the red samples showed an increased Ct-value above 29. Two samples from the < 5 ng/µl samples showed a Ct-value below 29 and thus, despite the low concentration, achieves a similar CT-value as the samples > 5 ng/µl. Overall, the red samples showed significantly higher Ct-values than the green samples (***p = 0.0004) and show limiting concentrations for RT-qPCR analysis.

**Figure 7:**
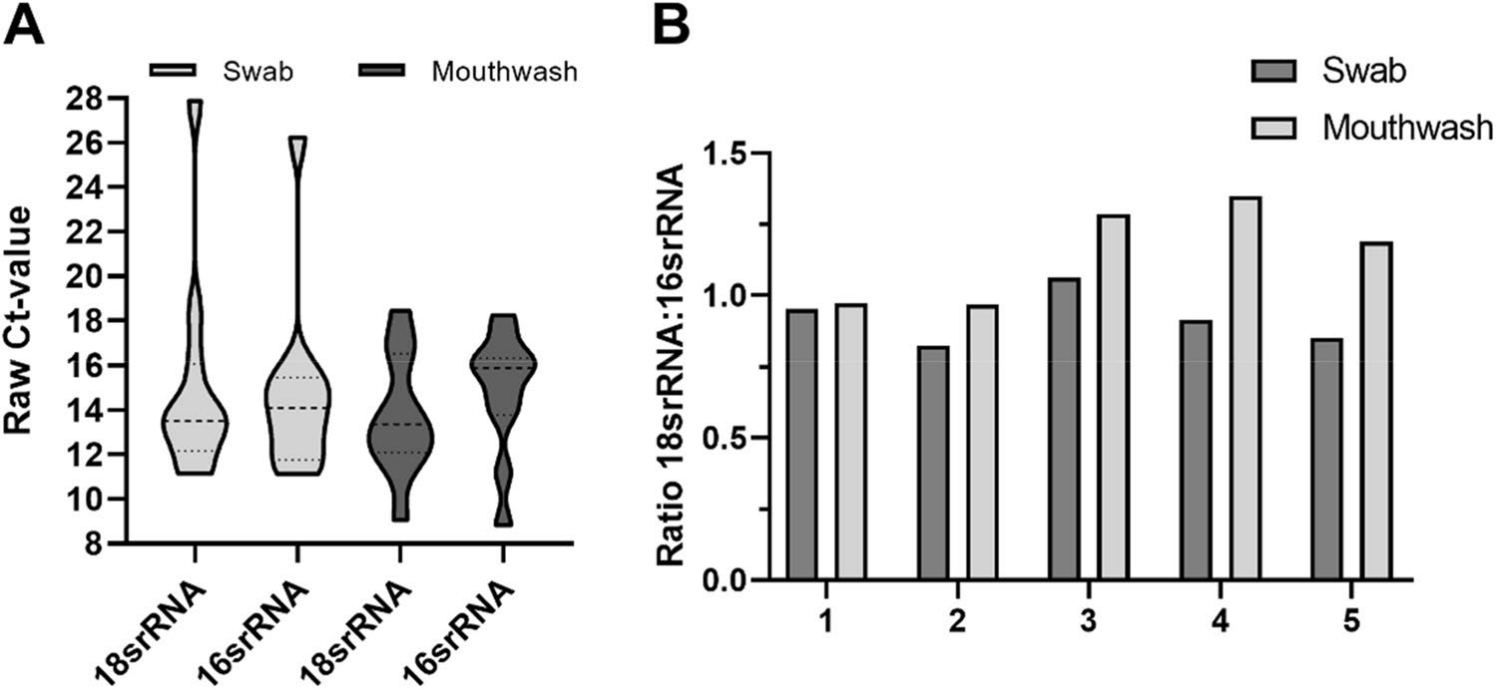
Isolation of bacterial nucleic acid. The violin plot in (**A**) displays the human *18srRNA* and bacterial *16srRNA* raw Ct-values for swab (n = 7) and mouthwash samples (n = 7). The bar graph in (**B**) shows the ratio of raw Ct-values (human *18SrRNA* and bacterial *16SrRNA*) of swab and mouthwash samples (n = 5). Samples were selected for their strong concentration deviation between swab and mouthwash (swab low, mouthwash high).

In order to determine the ratio between bacterial and human RNA in the samples and to be able to compare between the swabs and mouthwashes, the variable region 6 (V6) of the *16srRNA* gene was also analyzed for swab and mouthwash samples (n = 7) with RT-qPCR in addition to the *18srRNA* gene. The raw Ct-values are visualized in violin plots (Fig. 7A). Similar distributions are shown between 18 and *16srRNA* for the respective samples and the ratio is balanced. On average, the mouthwashes showed *16srRNA* Ct-values that are about two cycles higher than the swab samples.

### Parallel isolation of bacterial nucleic acid

In Fig. 7B samples, (n = 5) are selected again for verification, where the concentrations of the samples differ greatly between the methods. The mouthwash concentrations are min. sevenfold and maximum 50-fold higher than swab concentrations. Fig. 7B confirmed the balanced ratio of human and bacterial RNA in the samples, as already seen in Fig. 7A. On average, Ct-values of 16.778 (SD ± 4.779) for *16srRNA* and 15.792 (SD ± 6.126) for *18srRNA* were measured for the swabs. For the mouthwashes, the mean value was 14.062 (SD ± 2.872) for *16srRNA* and 12.125 (SD ± 1.623) for *18srRNA*. There were no significant differences between the *16srRNA* values (*p* = 0.501) indicating that the high concentrations of the mouthwashes compared to the swabs were due to increased bacterial RNA.

### Library and RNA sequencing quality control

**Figure 8:**
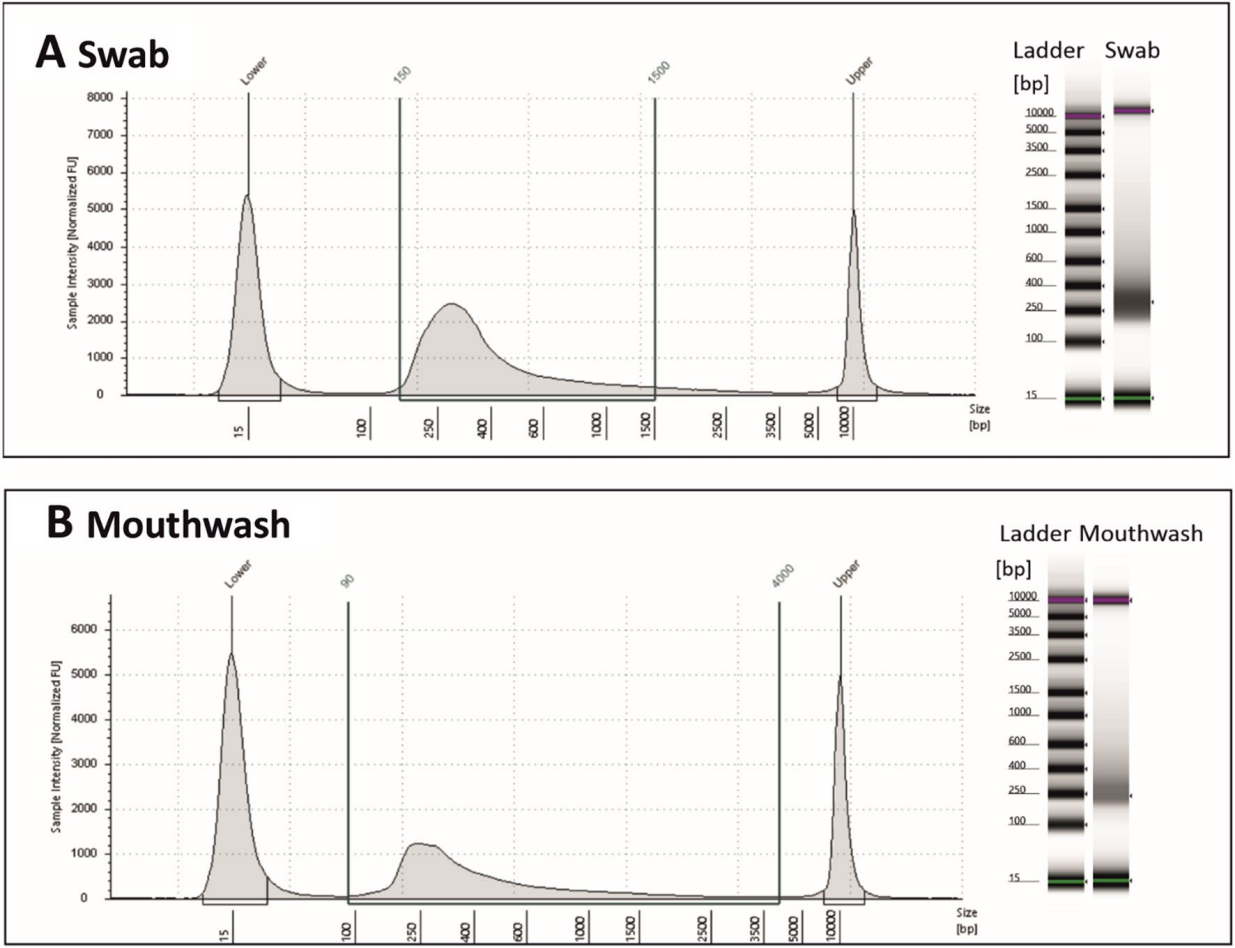
Library control with D5000 determination. In this figure electropherograms of D5000 analysis with Agilent Tape Station are shown. On the right side are the corresponding virtual gels with ladders from 15 to 10,000 bp. (**A**) shows the electropherogram of a swab sample with an average library size of 414 bp. Additionally, (**B**) shows a mouthwash sample with an average library size of 516 bp. The average size of approximately 500 bp fits the protocol being carried out. The electropherograms are without any additional peaks that could indicate primer or adapter dimers.

To check the quality of the library, we carried out a D5000 determination by Agilent TAPE station. Figure 8 shows electropherograms for a representative swab and a mouthwash sample. Electropherograms with the desired average size of approximately 500 bp are shown, additional peaks are not present.

Since the Q30 value is an important quality parameter for assessing the error rate for RNA sequencing, we looked at it comparatively for the swabs and the mouthwashes. Values of > 89% to 91% were found for both methods (raw data not shown). Another routinely used value to assess quality was generated with the software FastQC the “per base sequencing quality” (Suppl. Fig. 11). It shows an overview of the range of quality values across all bases at each position in the FastQ file. Differences between the samples are seen in the read length. In the mouthwash samples, the reads were about 20 bp shorter than the reads of swab samples. Most of the reads of swab samples had quality scores close to the maximum for Illumina sequencing. In mouthwash samples, some reads show poor quality calls as well as very good quality calls. However, quality of both of them were characterized as sufficient for downstream analysis.

## Discussion

### Sample composition and quantity

Simple and less invasive sampling methods for molecular biology analysis play an important role, especially in the oral cavity. In the future, molecular analyses could play a major role in the detection of pathogens but also for the diagnosis of cancer and inflammatory diseases.

For this reason, we have created and optimized a workflow that covers everything from sample collection and nucleic acid extraction to molecular analysis using RT-qPCR and RNA Sequencing. The aim was to develop a suitable method for different and varied downstream analyses and to describe a general handling process that is suitable for any subsequent analyses and particularly attractive for diagnostic procedures. To facilitate this aim, we have chosen cytobrushes and mouthwashes, a simple and comfortable method for both patients and doctors ^23^. We performed DNA and RNA isolations and quality analyses with n = 59 samples each. Our optimization strategies of the protocol according to the manual were particularly successful in the RNA isolation of the mouthwashes. The optimizations increased the concentrations for mouthwash samples by an average factor of 10. It was also observed, that the mouthwashes showed more consistent results than the swabs (Fig. 3B). This can be attributed to the better reproducibility of rinsing the mouth with NaCl for a specific time, rather than having different doctors take swabs. We observed also, that swab collection was dependant on the cooperation and of the patients, which would account for fluctuating values of isolated nucleic acids.

Similar to other studies, we were able to get an overview of the composition of the cells in the different materials by making cytological preparations of the swabs and mouthwashes^24,25^.

The comparison of the presence of immune cells is particularly interesting. Very few to no leukocytes were detected in the swab smears by microscopy. Mouthwashes, however, clearly showed the presence of different bacteria and leucocytes (Suppl. Fig. 1B). This is particularly interesting with regard to the nucleic acid concentrations, as we were able to achieve consistently higher concentrations with the mouthwashes, which can be attributed to the additional leucocytes contained in them. With a 40 µm cell strainer, we succeeded in separating the epithelial cells from the leucocytes (Suppl. Fig. 1C). With this sampling processing step, downstream analyses could be carried out on both cell types. With even smaller filters, it might also be possible to filter out bacteria and analyze them separately. Another possibility for separation could be density centrifugation. As a conclusion, one should be clear about the complex cellular composition of the mouthwash as source material and possibly initiate additional separation steps and use specific primers.

We were able to count more mucosal cells when a mouthwash was performed before the swab (Suppl. Fig. 2). In addition, we were able to detach more cell clusters after rinsing, which could not be counted at all during cell counting, which means that the number of cells after rinsing should be even greater. One potential explanation for the increased number of cells in the swab (after rinsing the mouth with NaCl solution), could be the mechanical loosening of mucosal cells by rinsing back and forth, or a combination of both. For this reason, we have always performed the mouthwash before the swab. For a diagnostic application, it is therefore also advisable to rinse the mouth before the swab to further increase the RNA yield. We compared our values with similar studies using different source materials. After optimization, we were able to isolate about 2.9 µg RNA for the mouthwashes and 0.9 µg RNA for the swabs. This amount is about 1.5 times more than compared with another study, in which RNA was also isolated from mouthwashes^26^. In the work from Sullivan et al., different RNA isolation kits of the company Zymo research and Qiagen were compared. In this study, saliva was used and the best concentrations of 6.8 µg were obtained with Zymo Directzol and 2.1 µg with the Quick Prep RNA Kit^27^. Thus, our concentrations are in the middle of these values, but it should be noted that different source materials cannot be directly compared accurately. However, a direct comparison is possible with the work of Kupfer et al., as they also performed swabs with cytobrushes of buccal mucosa and isolated RNA. They came up with an average value of about 0.37 µg, which is less than half the concentration we achieved with the swabs and our optimized method^28^. Another comparable study with cytobrushes shows the increase in yield by performing two swabs and pooling compared to one swab. However, two swabs only achieved concentration close to that which we achieved with one swab^29^.

Our patient cohort was divided into three different groups based on their symptoms and disease status, we also looked at the distribution of RNA concentrations within the groups. Since there were no observable differences (Suppl. Fig. 5), we conclude that the yield of nucleic acid seems to be independent of the clinical disease situation.

### Quality

We were able to show that increased protein contamination is not to be expected with the mouthwashes compared to the swab, seven though the source material was highly contaminated with glycoproteins. We achieved an average value for A260/280 ratio of about 1.9 with RNA samples of both methods, which is close to the optimum value of 2.0. Similar values from 1.5 to 2.0 have also already been measured with saliva samples^26,30^. The mean value of the measured A260/230 ratios for estimating the ethanol and salt contamination was about 1.8 for the RNA samples. We also determined RIN values for the RNA samples of the swabs and mouthwashes (Suppl. Fig. 6), which range from 1.1 to 5.3 and show rather degraded RNA for both sampling methods. Additionally, we performed a DV200 determination, which is a useful tool for classifying RNA according to its size distribution. For degraded RNA with a low RIN value, as is often the case with FFPE material, the DV200 value is determined as an additional quality measure, as the fragment distribution can have a major influence on the library yield of the sequencing ^31^. The values of our DV200 determination of both methods range from > 50 to  > 90% and are located in the medium or high range, showing a high percentage of fragments with > 200 nt. In this case, we did not observe any correlation between the RIN value and the DV200 values of the same samples, which is consistent with the fact that DV200 determination is superior to RIN analysis, especially for low-quality RNA, also with regard to the subsequent library quality^31^. Therefore, we would recommend, similarly to FFPE samples, to perform a DV200 determination also for RNA from the oral region.

In summary, it should be mentioned, that the fragmentation of RNA is frequently observed within oral specimens^32,33^, and do not preclude analysis by common molecular applications including RT‐qPCR^28^. To reduce the degradation it is important to use an appropriate normalization method with HKG^34^ and for downstream sequencing analysis an additional DV200 determination could be beneficial.

### cDNA synthesis

For the cDNA synthesis, we selected hexamer primers. Often, cDNA syntheses are performed with oligo-(dt) primers for human specific RT-qPCR. Oligo(dT) primers first bind via T: A base pairing to the poly(A) sequences present at the 3′-end of almost all mRNAs. The reverse transcriptase then extends from the annealed oligo(dT) primer along the mRNA template. This transcribes the mRNA sequence to the cDNA^35^. However, since at this point, a human-specific cDNA synthesis would exclude subsequent pathogen detection. In addition, especially at lower concentrations, cDNA synthesis using oligo-(dt) primers leads to a significantly lower yield and the resulting frequent need for preamplification, which could make quantification more difficult^36^. The additional step of pre-amplification is not a reliable method for all genes, and for some genes an amplification bias must be expected, which is due to low copy number in the starting material^37^. For our swab samples, we were also able to measure an average loss of two cycles in the Ct-value after cDNA synthesis with oligo-dT primers, compared to synthesis with hexamer primers. This supported our assumption that a higher yield can be achieved with hexamer primers (Suppl. Fig. 7). This shows the advantage of random hexamer primers compared to the use of oligo-(dt)-primers. The choice of priming strategy can have profound effects on the yield of cDNA synthesis. However, the yield also depends on the individual genes^38^.

### Bacteria isolation

To get information about the bacteria: human cell ratio in oral samples, we used the detection of *16srRNA* and *18srRNA* (Fig. 7). Raw Ct-values of both methods were located in similar ranges with both genes. This is an interesting observation in relation to the cell composition and the increased presence of bacteria in the mouthwashes, as well as the sometimes significantly higher RNA concentrations in the mouthwashes compared to the swabs of the same person. It would have been expected that the Ct-values of the *16srRNA* gene of the mouthwashes would be significantly lower than those of the swabs, but *16srRNA* Ct-values of swabs are about two cycles below the mouthwashes. This may lead to free bacterial RNA in the swab samples, which may have been depleted in the mouthwashes by the centrifugation and washing step with PBS. Since some samples showed a strong difference in the concentrations of mouthwash and swab of the same person, we initially suspected an increased bacterial: human cell ratio. Different oral hygiene conditions could be a reason for increased bacterial occurrence and could have explained an increased RNA concentration.

However, we were able to disprove this (Fig. 7B). We have assumed, that swab sampling is dependent on the person performing it and the individual density of immune cells, and this could be a possible reason for the low swab concentrations compared to mouthwash samples of the same patients. Individual patient tolerance and oral/pharyngeal sensitivities also play a role. The protocol was not optimized to extract bacterial RNA but, it was possible to derive microbial RNA from both sample types. A comparative study, using human saliva, showed an increased presence of bacterial RNA compared to human RNA. There were on average about 2.7 times more bacterial RNA copies compared to human RNA in total saliva^13^, which may have advantages in pathogen detection, but disadvantages for the detection of human transcripts compared to our method.

By quantifying the exogenous reads, they were also able to determine that 90.4% of the species in the saliva were bacteria. These include *Rothia mucilanginosa* (16,711 RPM), Rotia aeria (7605 RPM) and Streptococcus sanguinis (7136 RPM), which belong to the majority of bacteria found in human saliva^39^. Another study demonstrated that over 70% of cases showed the presence of more than 5% microbial contamination out of one to up to six species in a single sample. Interestingly, they were able to show that neither low, medium nor high contamination with non-human mapped reads had an impact on the clinical molecular diagnosis^40^. One way to improve the exRNA profile in saliva is to remove bacterial rRNA^41^. It has already been shown that selective removal of bacterial rRNAs with a commercial removal kit (Ribo-Zero™ Magnetic Kit) leads to an increase in sensitivity in the detection of human transcripts and genes (almost 50%) and could therefore be advantageous for performing analyses on human cells from oral samples^42^.

### Housekeeping gene analysis and qPCR limits

To test the usability of the samples for molecular analyses such as RT-qPCR and to be able to make a statement about the expression of different genes, we tested several frequently used HKGs for swab and mouthwash samples. HKGs play a major role in comparing and quantifying RT-qPCR data^43^ and a poor choice of HKG can lead to errors in the interpretation of experiments quantifying gene expression^44^. To test different HKGs for individual projects and materials, there are different software packages based on algorithms to perform the validation of reference^45^. Several comparable studies have already been published on HKG testing for human oral samples. For extracellular RNA from saliva, *RSP9*, *ACTB* and *GAPDH* were compared in the work of Feng Li et.al ^46^. *UBC* and *HPRT* were also found to be suitable HKGs for saliva by NormFinder for various states of the submandibular glands, for example, inflamed or atrophic states^47^. Other HKGs were already tested in saliva with the aim of detecting stable HKG within and between cancer and control groups. The most stable genes in this study were *ATP6*, *RLP30*, *RPL37A*, and *RPS17*^48^. These literature-based results emphasize the need to identify robust reference genes for specific applications. We therefore tested seven already in human (oral) samples using housekeeping genes and analyzed the measured values using the software NormFinder^14,28^. Stability values are calculated, that are inversely proportional to the actual stability of the gene. On the basis of the stability values, a ranking could be created for both methods (Fig. 5).

A comparative housekeeping gene analysis with saliva samples was carried out by Ostheim et.al. Interestingly, we see that with random hexamer primers we can obtain Ct-values of the HKGs that are in the same size range as with 14x-preamplified samples, and without preamplification, they could only achieve significantly higher Ct-values^13^.

*GAPDH* is one of the best known HKG, and used in for various analysis^49^. Supplementary Figure 8, shows that *GAPDH* is not suitable for our sample material due to a double peak in the melting curve, which is due to bacterial contamination, because the same primer shows no anomalies with pure human cell culture. This underlines the importance of melting curve analysis^44^.

For both methods, *TBP* was found to be the best HKG. Nevertheless, the ranking of the best genes for the respective method shows deviations with regard to the order of the best three genes (*YWHAZ, TFRC, TBP*). This shows that even with very similar samples and achieved Ct-values, different genes are better or worse suited, and that normalization and quantification can be optimized by improvement and optimal selection of HKGs. In Suppl. Fig. 9 we could show the stability of normalization on the target gene *BACE2* between the two best HKGs for the mouthwash samples (*TBP* and *TFRC*). The relative expression values showed almost no changes. Therefore, both HKGs could serve well for the normalization of target genes. For the swabs, however, there is a slightly greater difference, but the ratios of the rel. expression values still fit. Based on these results, we would suggest to use the *YWHAZ* shown by NormFinder as the best HKGs for normalizing the swabs samples,

Despite optimization as shown in Fig. 1, some samples gave values below 5 ng/µl, we asked ourselves to what extent these samples were nevertheless suitable for RT-qPCR analyses and could be used in molecular diagnostics. We therefore selected five samples with concentrations below 5 ng/µl to compare their HKG Ct-values. Three of these samples were already in the range of Ct-values of the negative controls carried and therefore appear to be critical for diagnostic purposes (Ct-value above 29). Not only low concentrations but also the presence of inhibitors can influence RT-qPCR results^50^. As can be seen from the values > 5 ng/µl, values with higher concentrations are also sometimes at higher Ct-values. Ct-values of two of the five samples were interestingly in the range of values above 5 ng/µl. This shows that 5 ng/µl can and should be used as a guideline for assessing the samples and their further analysis, but should not be seen as an absolute criterion for exclusion.

### Library and sequencing quality control

It is already recognized that samples with low RIN values can negatively affect molecular analyses, by impairing the detectability of targets^51,52^ and introducing bias^53^. RNA integrity has a significant impact on molecular analysis applications^52–54^ and is increasingly being investigated for its impact on transcriptomic analyses. We have already shown that RT-qPCR analysis is a suitable tool for downstream analysis of oral RNA samples like swabs and mouthwashes. Our aim was to check whether RNA sequencing is a possible downstream analysis of oral RNA samples too, despite their poor quality. We constructed a poly(A)-selected library, which is a common method for expression profiling and transcript quantification. We were able to show that it is possible to perform transcriptomic analyses using RNA sequencing with the Illumina NextSeq2000 platform with samples derived by our method. An essential first step is quality analysis of raw NGS data. To assess the quality of RNA-Seq, we used three routinely used quality parameters, the library control (D5000), phred score (Q30%) and the base per sequencing quality, which was satisfactory. We could show Q30 values of > 89% to 91%, which matches the Q30 value of RNA sequencing of body fluids, including buccal swabs with 89% of the total sequence of a quality above Q30^55^.

All our sequenced samples were below the recommended minimum threshold for RIN of 8 for RNA-Seq^56,57^. It is already known that although low RIN can still result in high sequencing performance, it can reduce library complexity and can impair transcriptome coverage^58^. In contrast, Lin et.al showed that no correlations were found between sequencing lead and the degree of degradation^55^. So far, the literature has mainly described DNA sequencing in oral samples. In a comparison of whole genome sequencing (WES) of blood and saliva samples, it was shown that saliva delivers a high sequencing quality for WES on an ion platform ^59,60^. However, for RNA sequencing, microbial rRNA depletion could increase the proportion of human RNA-Seq reads by approximately 30%, and therefore appears to be an important step in improving RNA sequencing of oral samples ^42^.

In summary, found satisfactory sequencing quality in our samples despite low RIN values, which offers the possibility of RNA-Seq as a method for research on oral diseases as well as for diagnostic purposes for oral samples.

## Conclusions

We have shown in this work an optimized protocol and processing work-flow from the clinic / bedside to the laboratory bench for obtaining nucleic acids (both RNA and DNA) from the oropharyngeal cavity for downstream molecular diagnostics. We have directly compared two methods of obtaining cells from the oropharyngeal cavity, i.e. mouthwashes versus swabs. Mouthwashes were found to give the highest amount of quantity nucleic acid retrieval. The gene *TBP* was found to be the best candidate out of seven common HKGs tested, for both methods.

### Supplementary Information

Supplementary Figures.

## Data Availability

The datasets generated and analyzed during the present study are available from the corresponding author upon reasonable request.
