# Supplementary material for: Characterization of non-invasive oropharyngeal samples and nucleic acid isolation for molecular diagnostics
Source: Sci Rep. 2024 Feb 19;14:4061. doi: 10.1038/s41598-024-54179-6 (PMC10876689; doi:10.1038/s41598-024-54179-6)
Supplement: Supplementary file 1 — Supplementary Figures. [file 41598_2024_54179_MOESM1_ESM.pdf]

Supplementary Figures

Supplementary Figure 1: Microscopy and cell counting to determine the cellular composition of initial and purified samples.

Supplementary Figure 2: Swab cell counting.

Supplementary Figure 3: Comparison of protocol optimization for DNA samples.

Supplementary Figure 4: Molecular properties of DNA.

Supplementary Figure 5: Nucleic acid concentrations between groups of the cohort.

Supplementary Figure 6: Virtual gel of the RNA Integrity Analysis (RIN).

Supplementary Figure 7: Influence of oligo dT primers on CT values.

Supplementary Figure 8: Comparison of GAPDH melting curves of patient RNA obtained from human cell cultures.

Supplementary Figure 9: Normalization of the target gene beta-secretase 2.

Supplementary Figure 10: Low DNA concentrations.

Supplementary Figure 11: Per base sequence quality whisker plot: distribution of quality of bases all over the whole file.

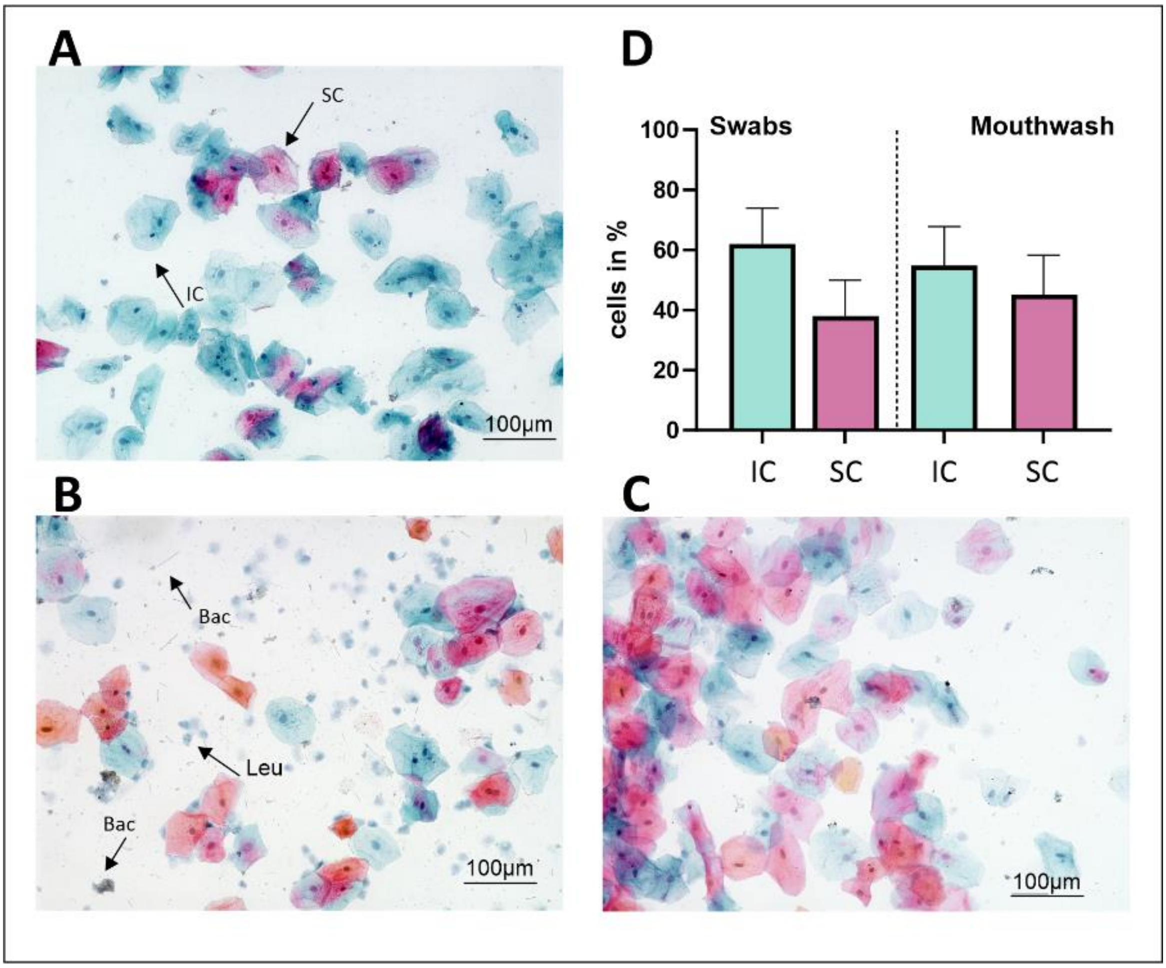

Supplementary Figure 1: Microscopy and cell counting to determine the cellular composition of initial and purified samples. (A-C) show microscopic images in PAP staining. (A) Buccal swab (B) Mouthwash (C) Mouthwash after 40 µm cell strainer. A compound of mucosal cells from different layers is shown in all samples. Both intermediate squamous cells (IC) and superficial squamous cells (SC) are stained. Relative cell counts of these cells are shown in (D), there is no significant difference between swab and mouthwash samples detected. In comparison to (A), (B) shows leukocytes (Leu) as well as different bacteria's (rods and spheres). As it can be seen in (C), we were able to filter out the leucocytes and bacteria with a 40 µm cell strainer to demonstrate the possibility to only analyze epithelia cells.

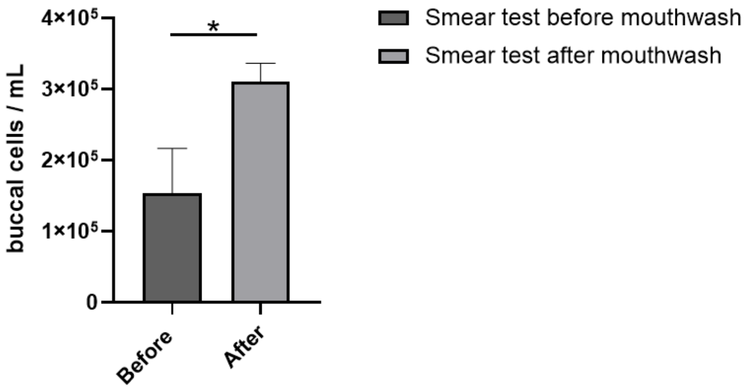

*Supplementary Figure 2: Swab cell counting. The order of swab and mouthwash sampling was tested. Cell counts of the swab samples were determined before and after mouth washes. Cell counts/ml are shown in bar plots. Significantly higher cell counts were obtained with the smear after mouth washing (t-test, two-tailed, 95% confidence interval, *p<0.05, **p< 0.01), (p *=0.0168).*

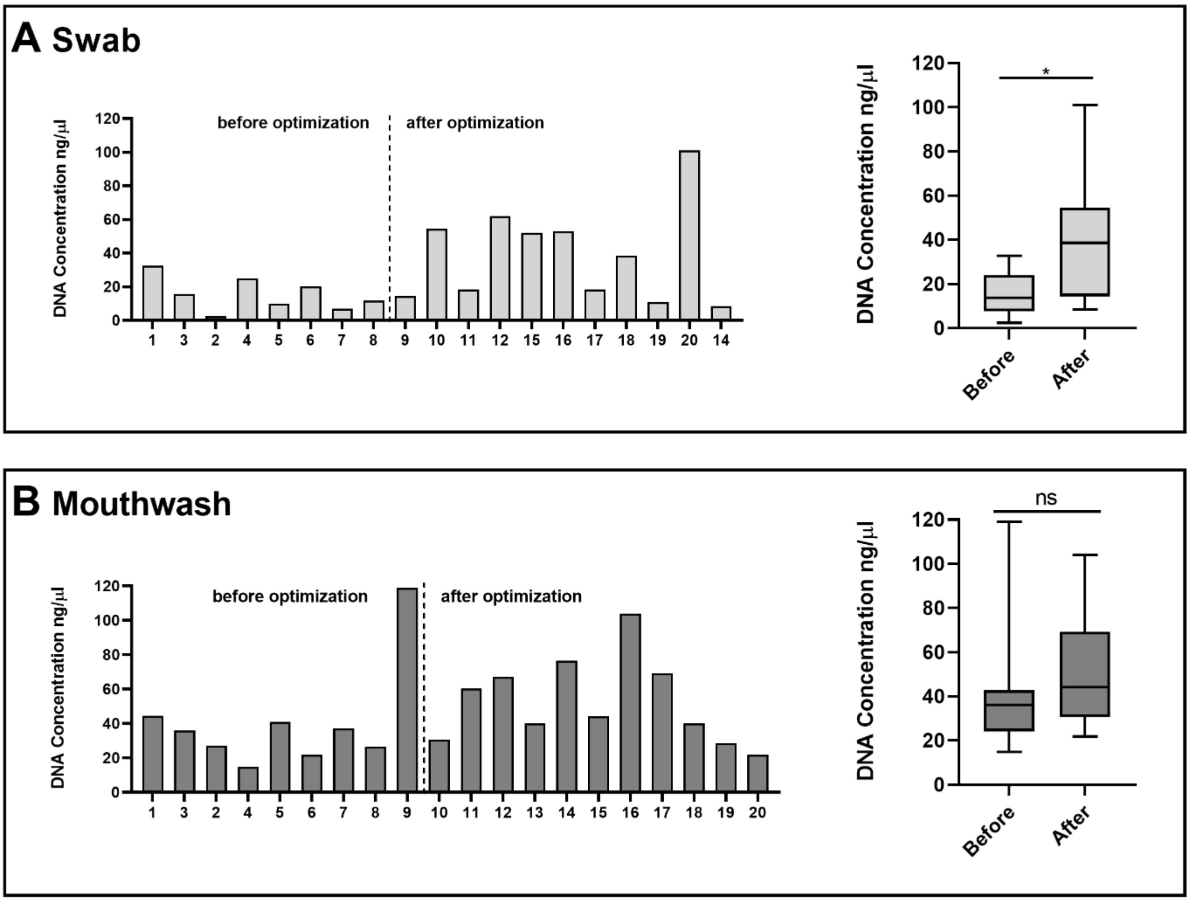

*Supplementary Figure 3: Comparison of Protocol optimisation for DNA samples. The bars on the left side in (A) and shows the DNA concentration of swab samples in ng/µl. The Figure shows the concentrations before and after protocol optimization, similar to Figure 3. The boxplots on the right side of (A) show same samples concluded into a boxplot for before and after optimisation. There is a significant difference between the boxplot's optimisation (t-test, two-tailed, 95% confidence interval, *p<0.05, **p< 0.01), (p =0.0402). Same graphs are shown for mouthwash samples below in (B). There is no significant difference between before and after optimization.*

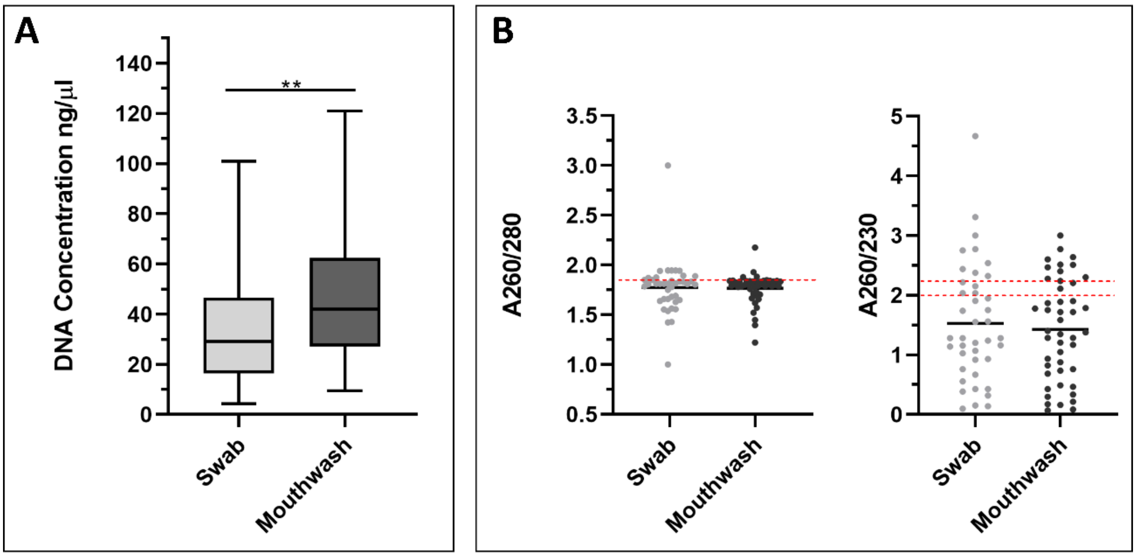

*Supplementary Figure 4: Molecular Properties of DNA. The boxplots in (A) show the amounts of total human and bacterial DNA in µg/µl extracted from the swab (n=39) and mouthwash (n=46) sample. There is a significant difference between swab and mouthwash DNA concentration (t-test, two-tailed, 95% confidence interval, *p< 0.05, **p< 0.01), (p** =0.0098). Quality of extracted DNA is shown in (B) for comparison, using the A260/230 and A260/280 ratios. Red lines show the area of "pure" nucleic acid. Swab and mouthwash samples show concerning values. The desired value of 1.8 for protein contamination is almost achieved on average by both methods with a mean of 1.775 for swab samples and 1.767 for mouthwash samples. The contamination of organic substances seems to be high for both methods, as the measured values were below the target value of 2.0-2.2. for the swabs a mean value of 1.529 and for the mouthwashes a value of 1.428.*

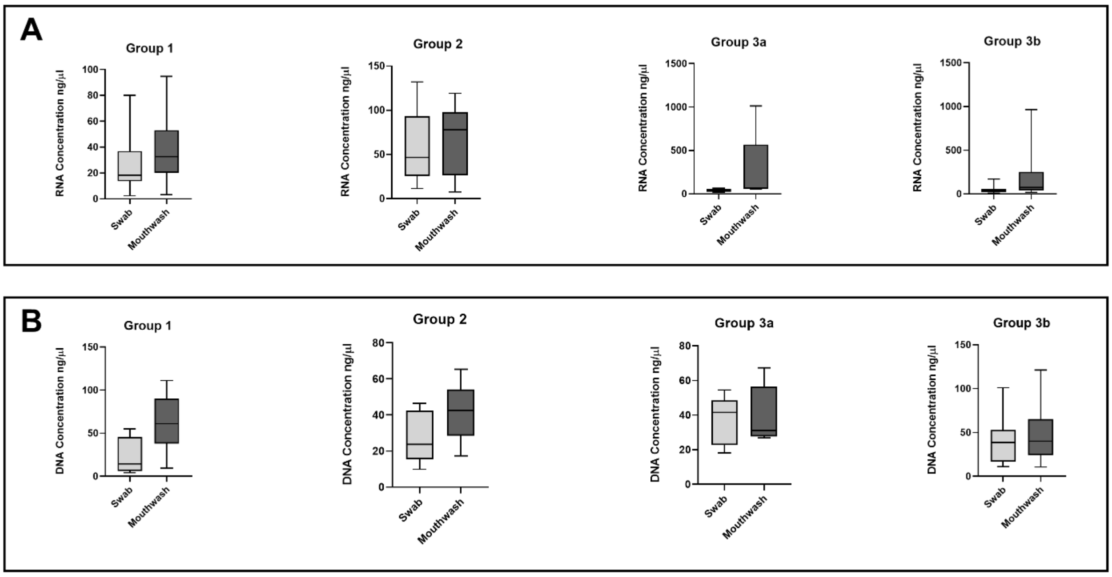

Supplementary Figure 5: Nucleic acid concentrations between groups of the cohort. The measured RNA and DNA values, are also divided into the different groups of the cohort in this figure. (A) shows the RNA samples and (B) the DNA samples of Group1, Group2, Group3a and Group3b. The main aim here was to check for differences in concentrations between the groups. Based on the boxplots shown, no correlation between a group of the cohort and the nucleic acid concentrations can be identified.

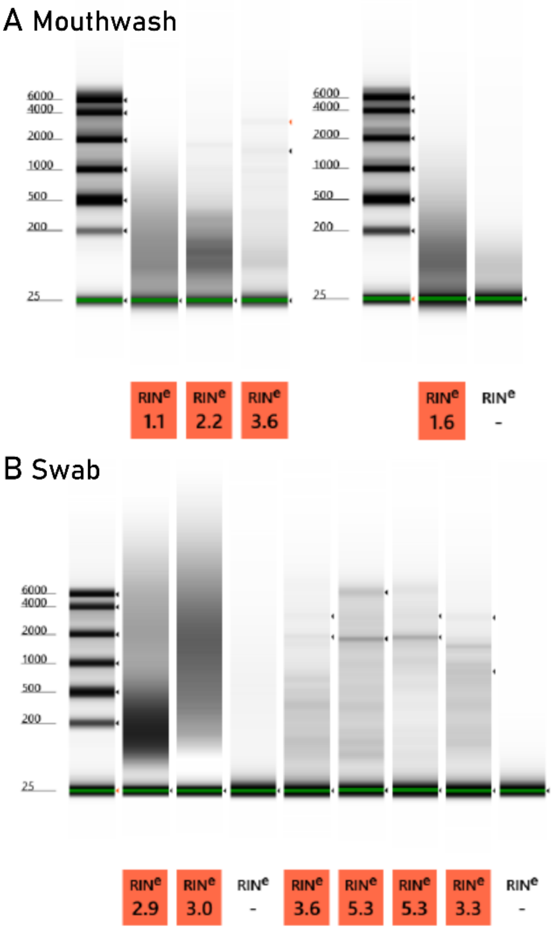

Supplementary Figure 6: Original Virtual Gel of the RNA Integrity Analysis (RIN). The ladder is shown on the left side with nucleotides ranges from 25 nt - 6000 nt. The RIN analysis of eight swabs and five mouthwash samples are shown. RIN values of about 3-5 were obtained for the swabs and values of about 1 -3.5 were measured for the mouthwashes. The RIN values obtained by both methods are low and thus indicate degraded RNA.

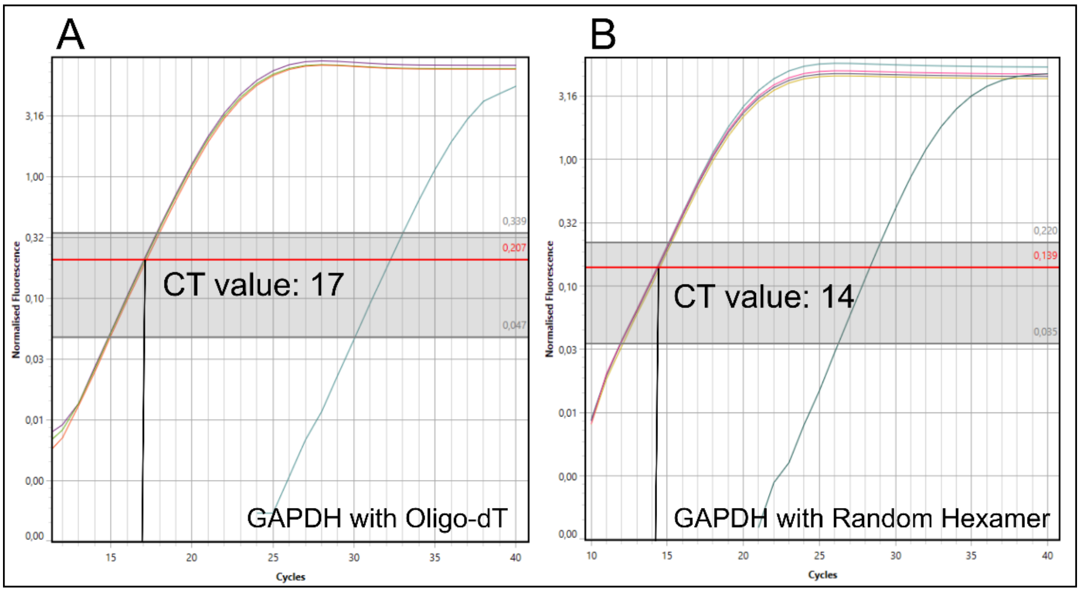

Supplementary Figure 7: Influence of oligo dT primers on CT values. A) shows qPCR curves generated with cDNA synthesized with oligo dT primers, B) with hexamer primers. A shows a Ct-value of 17 and thus indicates a lower cDNA yield compared to B, with a Ct-value of 14.

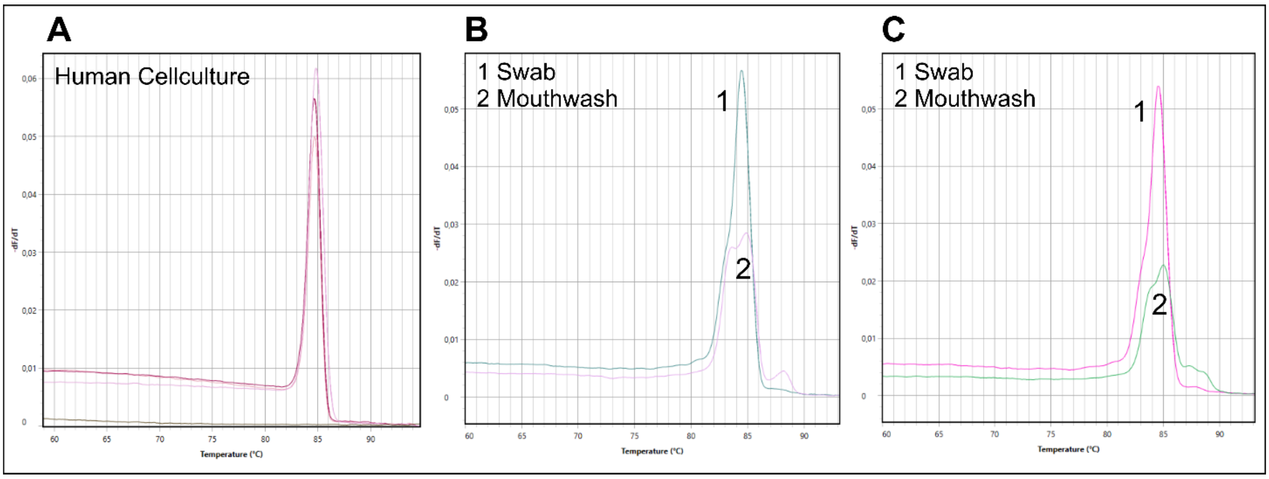

Supplementary Figure 8: Comparison of GAPDH melting curves of patient RNA obtained from human cell cultures. (A) shows the melting curves of a technical triplicate with the GAPDH primer and a human cell culture (a primary HNSCC cell culture). An exact peak is shown. (B) shows the melting curve of a swab (1) and mouthwash (2). The melting curves (2) in (B) show an unspecific double peak, which could due to parallel isolation by bacteria.

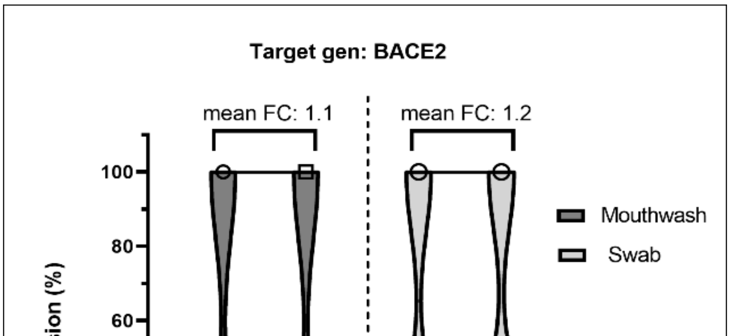

Supplementary Figure 9: Normalization of the target gene beta-secretase 2. The violin plots show the relative expression (%) of four mouthwash/ swab samples normalized to the two best HKGs ranked by NormFinder. Data points of same samples are connected with a line. Normalization of mouthwash samples with TBP and TFRC shows almost none differences in the relative expression values, while the values of swab samples differ more, but show also the expected ratio of expression values.

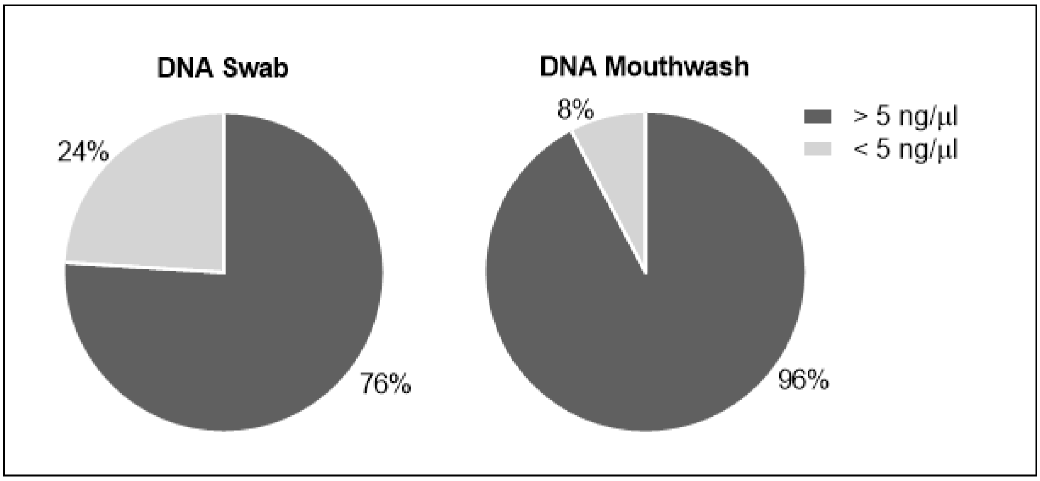

Supplementary Figure 10: Low DNA concentrations. The circle-chart presents an overview of the percentage of DNA concentration < 5ng/µl (light grey) and > 5ng/µl (dark grey) in swab and mouthwash samples. While 24% of swabs are under 5 ng/µl, only 8% of mouthwashes are under 5 ng/µl.

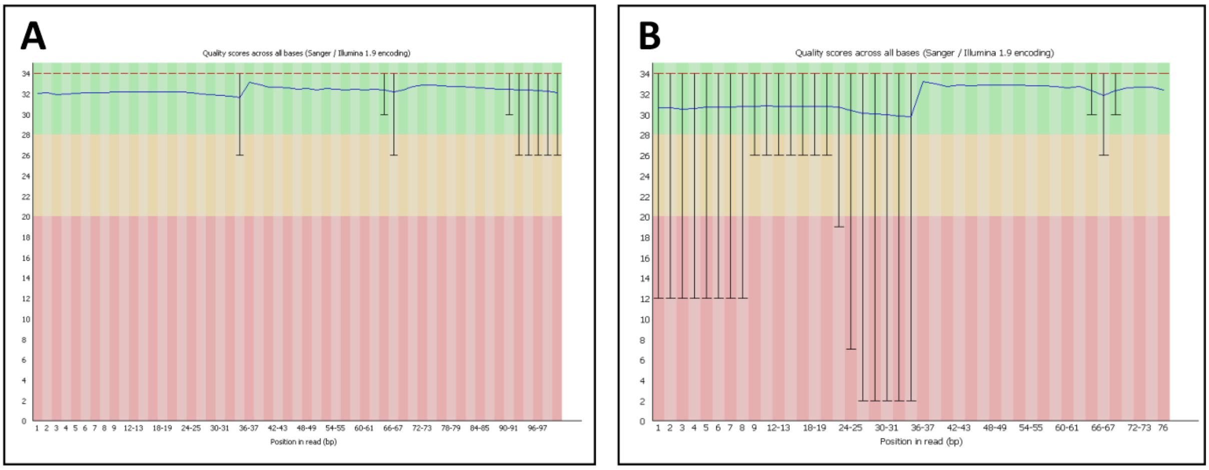

Supplementary Figure 11: Per base sequence quality whisker plot: distribution of quality of bases all over the whole file. An overview of the range of quality values for all bases at each position is illustrated. Only the error bars of the box plots are visible, as the values are close to the maximum and the interquartile range is in the upper green area. The y-axis shows the quality scores and the x-axis the position in read. The y-axis is divided into very good quality calls, reasonable quality calls and poor-quality calls. (A) shows the per base sequencing quality of a swab samples, in comparison to a mouthwash sample in (B). Quality of both of them were characterized as good by the software FastQC. Differences between the samples are seen in the read length, in the mouthwash samples the reads seem to be about 20 bp shorter than the reads of swab samples.
